# Correction: Salivary and serum interleukin-17A and interleukin-18 levels in patients with type 2 diabetes mellitus with and without periodontitis

**DOI:** 10.1371/journal.pone.0345302

**Published:** 2026-03-17

**Authors:** Suteera Techatanawat, Rudee Surarit, Kongthawat Chairatvit, Weerapan Khovidhunkit, Sittiruk Roytrakul, Supanee Thanakun, Hiroaki Kobayashi, Siribang-on Piboonniyom Khovidhunkit, Yuichi Izumi

In the Study population subsection of the Materials and methods, there is an error in the eighth sentence of the paragraph. The correct sentence is: Prior to research commencement and subject enrollment, the protocol was approved by the Institutional Review Board, Faculty of Medicine, Chulalongkorn University (IRB No. 100/57) and the Institutional Review Board of the Faculty of Dentistry/Faculty of Pharmacy, Mahidol University (MU-DT/PY-IRB 2011/012.3103).
